# The Effects of Unripe Grape Juice on Lipid Profile Improvement

**DOI:** 10.1155/2012/890262

**Published:** 2012-08-29

**Authors:** Mohammad Javad ZibaeeNezhad, Esmael Mohammadi, Mohammad Ali Babaie Beigi, Fatemeh Mirzamohammadi, Oveis Salehi

**Affiliations:** ^1^Department of Cardiology, Cardiovascular Research Center, Shiraz University of Medical Sciences, Shiraz, Iran; ^2^Cardiovascular Research Center, Faghihi Hospital, Shiraz University of Medical Sciences, Shiraz, Iran; ^3^Metabolic Disease Research Center, Zanjan University of Medical Sciences, Zanjan, Iran

## Abstract

*Introduction.* Consumption of unripe grape juice (verjuice) has been portrayed by the traditional belief, as a means of combating dyslipidemia. We aimed to evaluate the effects of unripe grape juice consumption on lipid profile in healthy human volunteers. *Methods.* We asked 42 enrolled volunteers to drink 10 cc of verjuice within 30 minutes to 2 hours after lunch and 10 cc of it after dinner. After taking 120 doses of verjuice, another fasting lipid profile was obtained from each participant. The statistical analysis was performed by SPSS 13 software. *Results.* After analysis of the data, the mean ± standard deviation for all the variables was obtained. Among those improvement of HDL-C was significant after the trial (*P* value < 0.001). TG, TC, and LDL improvement were not significant. *Conclusion.* Our study declared that verjuice has a dramatic effect on improving HDL-C level of serum but no any other lipid improvement effect was obtained.

## 1. Introduction

Despite the progress in prevention and early diagnosis, cardiovascular disease (CVD) remains the leading cause of death in both men and women throughout the world. No other life-threatening disease is as prevalent or expensive as CVD to society, affecting the majority of adults past the age of 60, and persons with CVD are likely to die from their disease [1].

Many of the important risk factors for cardiovascular disease are modifiable by specific preventive measures. Among these risk factors dyslipidemia is sorely important because of the major role in initiation and progression of atherosclerosis as the main reason of CVD [2, 3].

Unripe grape juice use (verjuice) in many ways is prevalent in Iranian families' meals which include mixing with different kinds of salads, for changing the taste of foods to sour, and even drinking some amount of it. This extensive use may be the reason of the general belief in Iranian traditional medicine that verjuice has some lipid-lowering effects [4]. Is it now reaping the fruitage of what it has sown for centuries? As we endeavor to this vital question, we found few studies conducted on verjuice, which have been done recently with extremely different conclusions.

Findings recently suggested that there is an inverse relationship between the intake of flavonoids and CVD mortality [5, 6]. Grapes contained many polyphenols, including flavonoids, flavanols, flavans, and anthocyanins [5, 6].

In vitro studies revealed that grape juice has significant antioxidant activity, which can reduce the level of oxidized LDL by inhibiting oxidation of low-density lipoprotein [7, 8]. They also declared that decreasing plasma concentration of LDL and increasing those of high-density lipoprotein can be achieved simultaneously [7, 8]. Although many studies have been done on grape juice which were greatly supportive of improving dyslipidemia, only a few of them have been conducted on verjuice. In addition, some studies do not support the improving effect of verjuice in dyslipidemia. Despite all these, the usefulness of verjuice on dyslipidemia is still unclear. Therefore, we conducted a prospective study on 42 dyslipidemic healthy volunteers to determine the paraclinical effects of verjuice on lipid profile modulate.

## 2. Method

All the subjects were recruited from Healthy Heart House of Shiraz Cardiovascular Research Center, a referral center in south of Iran. This study has been approved by the review board and ethics committee of Shiraz University of medical sciences (Ethics no. 2019). All the subjects who participated gave written informed consent before enrollment. The study population consisted of 42 volunteers with impaired lipid profile, who enrolled between November 1, 2011, and February 12, 2012. The subjects could be of either sex and without any race limitation.

To be included in the study, subjects had to have low baseline levels of HDL cholesterol (<40 mg per deciliter (1.03 mmol per liter) for men; <50 mg per deciliter (1.29 mmol per liter) for women), who were taking no medication, with no history of diabetes or any endocrine dysfunction. Subjects with a history of renal dysfunction, hepatic diseases, depression, and cardiovascular diseases and of course subjects without compliance were excluded.

A fasting lipid profile was obtained in eligible patients to establish baseline levels. Blood samples were collected and allowed to clot within 15 minutes at room temperature, then centrifuged at 1500 rpm in a bench centrifuge for 6 minutes. Analyses were performed by the core laboratory of Healthy Heart House by Biolis 24i Model PREMIUM device (japan).

The serum concentration of triglycerides was measured as glycerol after enzymatic hydrolysis with lipase/esterase (Pars Azmoon, GPO-TAP, Lot no. 90014). Total cholesterol was measured by enzymatic reagent (Pars Azmoon, CHOD-TAP, Lot no. 90016). HDL-C was measured by direct enzymatic method using Direct HDL cholesterol(IBD)(Pishtaz Teb, Lot no. 9002).

LDL-C levels were estimated by using the Friedewald formula: LDL-C = TC − (HDL-C + TG/5) [9]. All measurements were made under standard conditions by one technician and with the same device.

The unripe grapes were provided by arbors of Shiraz. They have been picked at the stage of total sourness. All the grapes were green, unripe, and sour at the time of picking. We separated the grapes from the stalk and poured them into big pans. Then we washed them with mineral water, and placed them in the dark for 5 hours on aluminium foils to evaporate the surface water. For producing the juice we used the Ahmadi's device in 400 round per minute which is specialized for grape juice. We poured the juice in 20-liter plastic containers. Maximal storage time of each container was 15 days. 300 cc verjuice had been given to the volunteers every 15 days in separate bottles. The verjuice was totally sour, and the colour was grass green at the time of giving to the subjects.

We asked the volunteers to drink 10 cc of prepared verjuice within 30 min till to 2 hours after lunch and 10 cc of it after dinner. The duration of study was 60 days for each individual.

All volunteers continued their habitual diets in the course of study and had to come to our center every 15 days for taking the next verjuice bottle. We asked them to explain about their experience in the period of using the verjuice. Two of the volunteers complained about epigastric pain which was mild, so they used the verjuice cooperatively.

After taking 120 doses of verjuice, another fasting lipid profile was obtained from each participant. All the obtained data were documented and transferred to SPSS ver. 13 software. The mean levels of TG, T-Chol, LDL-C, and HDL-C were analyzed using the paired samples *t*-test. All the results were reported as mean ± SD.

## 3. Result

Of 42 individuals, finally enrolled in the study, 7 were excluded because of epigastric pain or low compliance. Of the 35 subjects, 54% were men. The mean age was 43.3 years (range, 24 to 58), and the mean body mass index was 27.23 (range 17.1 to 48.6).

HDL-C values were 35.83 ± 4.40 mg/dL and 45.86 ± 4.30 mg/dL before and after verjuice consumption, respectively (*P* value = 0.001). Serum TG levels measured 237.28 ± 21.15 mg/dL and 211.48 ± 18.60 mg/dL before and after verjuice consumption, respectively, which shows 11% decrease, but it was not statistically significant (*P* value = 0.111). With respect to all recorded characteristics, Table 1 shows all the baseline and final study parameters.

The mean average of total cholesterol had 5% increased after the study and hit 200.34 mg/dL. LDL-C had about 5% rising which was not statistically significant (*P* value = 0.183).

## 4. Conclusion

This survey evaluated the lipid-lowering effect of unripe grape juice (verjuice) in humans. Based on the results of this study (95% confidence interval), verjuice had a significant increasing effect on serum levels of HDL, but no significant statistical effect on total cholesterol, LDL-C, and TG after 2 month of 20 cc daily verjuice consumption.

HDL-C is known to be a significant and independent predictor of CHD risk. A recent meta-analysis of four large prospective studies revealed that, by 2% increasing in serum HDL-c, the incidence of coronary events decreases by 2% in men and 3% in women [10].

Just a few studies were conducted on verjuice; one of them was conducted in our center by Aminian et al. which showed that unripe grape juice consumption had no incremental effect on serum HDL-C levels in healthy individuals. They also stated that verjuice has no effect on TG, total cholesterol, and LDL-C [11]. A major drawback of that study was the very high amount of verjuice (80 cc daily in one ingestion after lunch) which is rarely compliable. They also did not mention producing methods and even the taste or color of the verjuice which is very important. In another study conducted in 2006 by Aminian et al. on 5 groups of rabbits, they induced hyperlipidemia in them by using 10 mL egg yolk daily and then they used verjuice in some groups and compared the lipid profiles among groups. That study, did not support preventive or therapeutic effect of verjuice in dyslipidemia [4]. The authors of this study also refrained from mentioning the quality of verjuice.

On the other hand Khadem-Ansari et al. showed that red grape juice is effective in increasing HDL [12]. In addition ingestion of concentrated red grape juice as a polyphenol-rich dietary supplement exerts hypolipidemic, antioxidant, and anti-inflammatory effects [7]. Additionally Stein et al. revealed that purple grape juice consumption improved endothelial function and reduced the susceptibility of LDL-C to oxidation in patients with cardiovascular disease [13].

Moreover, in a study on hamster model which accomplished by Vinson et al. they found out that grape juice was much more effective than red wine or dealcoholized wine at the same polyphenol dose in inhibiting atherosclerosis and improving lipids and antioxidant parameters. They indicated that polyphenolic beverages from grapes are beneficial in inhibiting atherosclerosis by several mechanisms [14].

One of our limitations should be noted. The control group was not available for us to restrict the regression to the mean effect.

In conclusion, our study revealed that unripe grape juice has a dramatic effect on improving HDL-C level of serum but no lipid-lowering effect on TG, total cholesterol, or LDL-C. According to the results of this study we could conclude that regular consumption of unripe grape juice has a dramatic effect on improving serum level of HDL-C but TG, total cholesterol, and LDL-C are not significantly affected. Slightly increased total cholesterol in the results of this studyis may because of increased HDL-C level. Although the aforementioned studies have used red grape juice as an intervention that caused lipid profile improvement, we claim that verjuice could play a better effect on HDL-C level, but still further investigation is needed.

## Figures and Tables

**Table 1:** Lipid indexes before and after the study and their comparison.

Lipid indexes	Before	After	*P* value
TG	237.28 ± 21.15	211.48 ± 18.6	0.111
HDL	35.83 ± 4.4	45.86 ± 4.3	0.001
LDL	108.58 ± 12.4	114.79 ± 11.8	0.183
Chol	190.46 ± 17.3	200.34 ± 17.7	0.031
